# Assessing microvascular dysfunction and predicting long-term prognosis in patients with cardiac amyloidosis by cardiovascular magnetic resonance quantitative stress perfusion

**DOI:** 10.1016/j.jocmr.2024.101134

**Published:** 2024-12-14

**Authors:** Leting Tang, Wenjin Zhao, Kang Li, Lin Tian, Xiaoyue Zhou, Hu Guo, Mu Zeng

**Affiliations:** a Department of Radiology, The Second Xiangya Hospital, Central South University, Changsha, China; b Circle Cardiovascular Imaging Inc., Changsha, China; c MR Collaboration, Siemens Healthineers Ltd., Shanghai, China; d MR Application, Siemens Healthineers Ltd., Changsha, China; e Clinical Research Center for Medical Imaging in Hunan Province, Changsha, China

**Keywords:** Cardiac amyloidosis, Microvascular dysfunction, Stress CMR, Prognosis

## Abstract

**Background:**

Cardiac involvement in light chain amyloidosis (AL) is the main determinant of prognosis. Amyloid can be deposited in the extracellular space and cause an increase in extracellular volume fraction (ECV). At the same time, amyloid can also be deposited in the wall of small vessels and cause microvascular dysfunction. This study sought to investigate the extent of microvascular dysfunction and its incremental prognostic value in cardiac light-chain amyloidosis (AL-CA) by quantitative stress perfusion.

**Methods:**

A total of 126 AL amyloidosis patients (61.13 ± 8.46 years, 81 male) confirmed by pathology were prospectively recruited. All subjects underwent cardiovascular magnetic resonance (CMR) with late gadolinium enhancement (LGE), T1 mapping, and stress perfusion on a 3T scanner. ECV and myocardial perfusion reserve (MPR) were measured semi-automatically using a dedicated CMR software. Clinical, laboratory, and CMR parameters were analyzed for their prognostic value in the assessment of AL-CA patients. Mortality-associated markers were analyzed by univariate and multivariable Cox regression.

**Results:**

The median follow-up time was 37 (33.6–40.4) months, and 62 patients died. The ECV of survivors was significantly reduced, but the stress myocardial blood flow and MPR were higher (P < 0.001). The MPR of the transmural LGE group was significantly lower than that of the no LGE and subendocardial LGE groups (P < 0.001). In multivariable analysis, ECV, MPR, and LGE were independently predictive. MPR of >1.5 and ECV of ≤53.6% were associated with improved overall survival, both of which provided predictive incremental value in patients with advanced disease. With equal Mayo staging and degree of ECV, MPR improves assessment of patient survival.

**Conclusion:**

ECV and MPR showed additive incremental values and further discriminated prognosis of patients in advanced stages. CMR phenotypes with higher ECV and lower MPR had a worse prognosis.

## Graphical abstract

**Figure:**
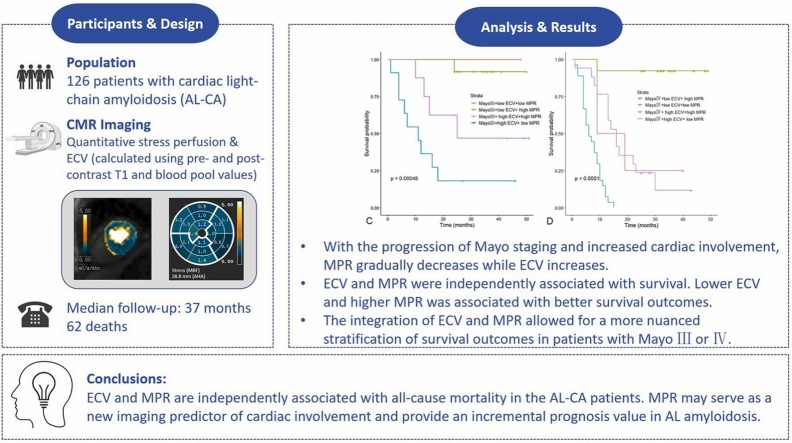

## 1 Introduction

Immunoglobulin light chain (AL) amyloidosis is characterized by monoclonal plasma cells and the deposition of insoluble amyloid fibrils formed by immunoglobulin ALs in various organs [1]. Approximately two-thirds of patients with AL amyloidosis have cardiac impairment, and cardiac involvement drives the prognosis [2], [3]. Early identification and risk stratification of individuals with cardiac involvement is critical. Cardiac amyloid is deposited in the myocardial extracellular space, disrupting normal tissue architecture and function. Notably, amyloid not only accumulates in cardiac walls, but also infiltrates perivascular and capillary vessels, leading to narrowing of the vessel lumen, capillary rupture and rarefaction, which then contributes to myocardial microvascular dysfunction [4].

Cardiovascular magnetic resonance (CMR) with tissue characterization is a sensitive tool for detecting myocardial amyloid deposition [5]. Native T1 and extracellular volume fraction (ECV) can be used to measure the deposition of amyloid in myocardial tissue and detect myocardial abnormalities earlier than late gadolinium enhancement (LGE), which is an important imaging indicator for the early diagnosis of amyloidosis [6]. In some studies, native T1 and ECV were independent predictors of mortality in patients with cardiac amyloidosis [7], [8]. However, limited studies have delved into microvascular dysfunction in amyloidosis. While previous studies have identified myocardial microvascular dysfunction in amyloidosis using qualitative or semi-quantitative myocardial perfusion techniques [9], the relationship between myocardial microvascular dysfunction and amyloidosis severity remains unclear without prognostic analyses. In addition, CMR advances enable assessment of myocardial perfusion by automated in-line perfusion mapping techniques allowing for pixel-level quantification of myocardial blood flow (MBF) and myocardial perfusion reserve (MPR), which provides a quantitative indicator for assessing myocardial microvascular dysfunction [10], [11]. Previous study has shown that cardiac amyloidosis is associated with severe inducible myocardial ischemia demonstrable by histology and CMR stress perfusion reduction [12]. However, to the best of our knowledge, few studies have investigated myocardial microvascular dysfunction in patients with cardiac light-chain amyloidosis (AL-CA) using fully quantitative stress perfusion CMR, and the prognostic value of stress perfusion CMR for AL-CA has not been thoroughly studied.

Therefore, this study aims to assess myocardial microcirculation in patients with AL-CA using quantitative stress perfusion techniques with pathologic validation and to evaluate whether the MPR can provide incremental prognostic value for further risk stratification in patients with AL-CA.

## 2 Methods

### 2.1 Study population

**Fig. 1:**
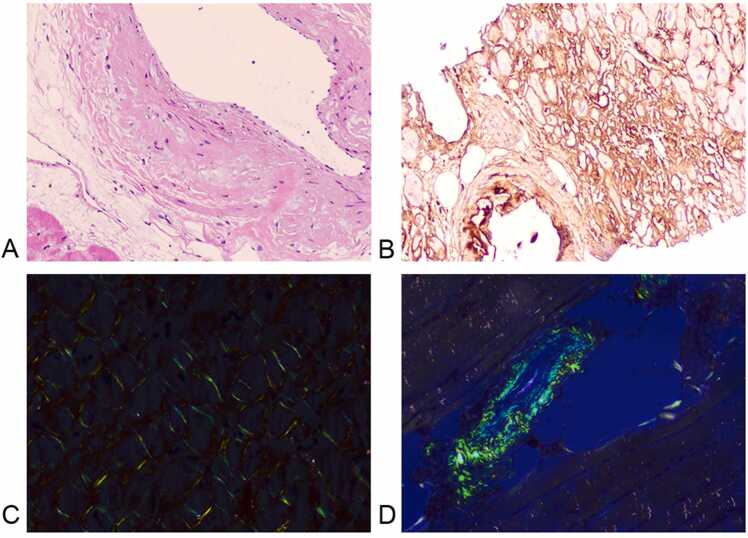
Endomyocardial biopsies. (A) Hematoxylin-eosin stain and (B) Congo red stain of cardiac tissue; (C) Congo red staining of myocardial biopsies showing green birefringence under polarized light; (D) shows amyloid deposits in small arteries along the entire circumference

This prospective study was approved by the Institutional Ethics Committee for the Second Xiangya Hospital of Central South University. All subjects have consented to participate in this study. AL amyloidosis patients who were referred for CMR imaging at the Second Xiangya Hospital between 2020 and 2022 were included in the study. One hundred and twenty-six patients with AL-CA (61.13 ± 8.46 years, 81 male) were consecutively recruited. All patients had biopsy evidence of AL amyloidosis with positive Congo red stain and AL deposition confirmed by immunohistochemistry (Fig. 1). Cardiac involvement was established by CMR by the presence of diffuse subendocardial or transmural LGE, altered gadolinium kinetics and/or diffusely elevated ECV. All patients underwent laboratory examination of the cardiac biomarkers troponin T and N-terminal pro-B-type natriuretic peptide (NT-proBNP), serum immunoglobulin free light chain difference (dFLC), fasting blood glucose, triglyceride, total cholesterol, and creatinine at baseline and were categorized based on revised Mayo Stage, as published in 2012 [13].

### 2.2 CMR imaging protocol

**Fig. 2:**
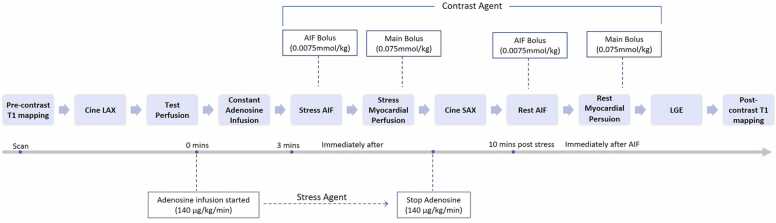
The CMR scanning protocol. *AIF* arterial input function, *LAX* long-axis, *LGE* late gadolinium enhancement, *SAX* short-axis

CMR imaging was performed on a 3T scanner (MAGNETOM Skyra; Siemens Healthineers, Erlangen, Germany) equipped with 18-channel body coil scan protocols. Electrocardiogram was monitored during the scanning to correct heart-rate–related rest and stress MBF. The scanning protocol is shown in Fig. 2.

Quantitative myocardial perfusion imaging was performed during stress and at rest, using a steady-state free precession pre-bolus adenosine protocol for quantifying absolute myocardial blood flow. Adenosine was infused at a constant state for 140 µg/kg/min for 3 min. At the 3-minute mark, stress arterial input function (AIF) acquisition was initiated under free breathing. After five baseline frames, the AIF bolus (comprising 90% saline and 10% contrast [Gadovist; Bayer HealthCare, Berlin, Germany]; contrast dose 0.0075 mmol/kg) was injected. This was followed by a flush with at least 20 mL of saline and the acquisition was continued for a total of 60 frames. Immediately after, stress myocardial perfusion acquisition under free breathing was started. After five baseline frames, the main bolus (0.075 mmol/kg) was injected. For further calculation information, please refer to : https://www.circlecvi.com/qp-protocol/index.html. Then, it was flushed with at least 20 mL of saline, and the acquisition continued for a total of 60 frames. After stress myocardial perfusion acquisition, adenosine infusion was stopped. A 10-minute wait was observed during which cine images were acquired. At the 10-minute mark, rest AIF acquisition was started under free breathing. After five baseline frames, the AIF bolus (0.0075 mmol/kg) was injected and flushed with at least 20 mL of saline. The acquisition was continued for a total of 60 frames. Immediately after rest AIF acquisition, rest myocardial perfusion acquisition was started under free breathing. After five baseline frames, the main bolus (0.075 mmol/kg) was injected and flushed with at least 20 mL of saline. The acquisition was continued for a total of 60 frames. After rest myocardial perfusion acquisition, LGE images were acquired.

Myocardial perfusion imaging was performed using a saturation recovery sequence based on fast low-angle shot imaging sequence. The scan parameters of first-pass perfusion imaging were repetition time: 2.0 ms; echo time: 1.0 ms; flip angle: 10°; field of view: 320 mm × 400 mm; and acquisition matrix: 125 × 256; and slice thickness, 8 mm, slice gap 2 mm.

Breath-hold cine imaging was performed using a segmented balanced steady-state free precession sequence. The scanning parameters were as follows: repetition time, 3.2 ms; echo time, 1.43 ms; flip angle, 44°; temporal resolution, 40 ms; field of view, 320 mm × 400 mm; acquisition matrix, 126 × 224; slice thickness, 8 mm with 2 mm gap.

LGE sequences were acquired in the same slice position as cine sequences. Scan parameters were as follows: repetition time: 2.8 ms; echo time: 1.3 ms; flip angle: 40°; field of view: 320 mm × 400 mm; acquisition matrix: 125 × 256.

ECV was calculated using pre- and post-contrast T1 and blood pool values according to the following formula: ECV = (1 − hematocrit) × [Δ(1/T1) myocardial/Δ(1/T1) blood flow]. Hematocrit was measured on the same days as the CMR examination. Pre- and post-contrast T1-mapping sequences used a modified look-locker inversion recovery method with 5b(3b)3b and 4b(1b)3b(1b)2b scheme, respectively. The scanning parameters of myocardial T1 mapping were as follows: repetition time: 277 ms; echo time: 1 ms; flip angle: 35°; field of view: 320 mm × 400 mm; and acquisition matrix: 125 × 256.

### 2.3 CMR analysis

**Fig. 3:**
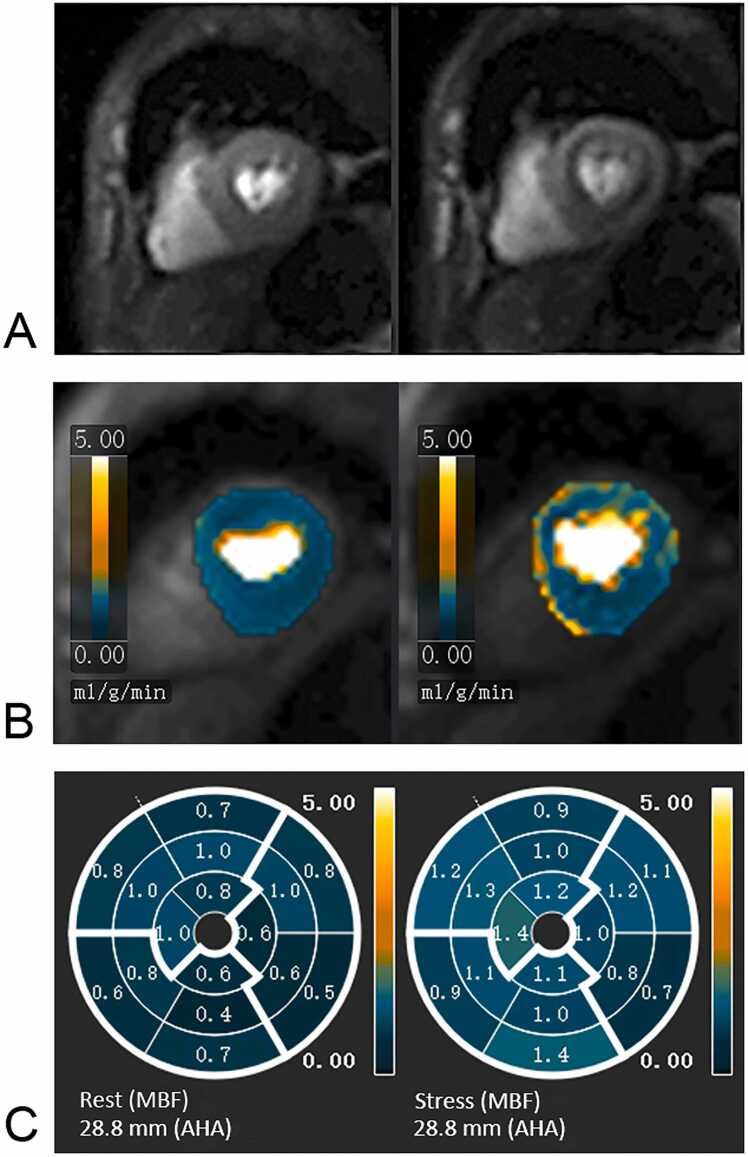
Quantitative stress perfusion CMR. (A and B) Automated MBF pixel maps in a patient show a perfusion reduction on the stress perfusion image and MBF map; (C) the bull's eye map of quantitative myocardial perfusion showing the rest MBF and stress MBF. *AHA* American Heart Association, *CMR* cardiovascular magnetic resonance, *MBF* myocardial blood flow

All CMR images were postprocessed by Circle software (Circle Cardiovascular Imaging Inc., Calgary, Alberta, Canada). CMR images were interpreted independently by two experienced radiologists who had more than 5 years of experience (H.G. and M.Z.). According to the American Heart Association (AHA) left ventricular segmental analysis, the myocardium was divided into 16 segments [14]. For quantitative analysis of MBF, the software provided a fully automated framework that includes a model-constrained deconvolution technique to estimate pixel-wise MBF values in mL/g/min by processing time-signal intensity curves [15]. If necessary, the auto-segmentations were manually adjusted. MPR was calculated as stress MBF divided by rest MBF. Global MPR was calculated as the average value of all 16 segments. Representative images are shown in Fig. 3. Function parameters, including left ventricle (LV) ejection fraction (LVEF), LV end-diastolic volume (LVEDV), LV end-systolic volume (LVESV), and LV mass, were measured from cine images. The LGE pattern was classified into three groups: (1) no LGE (absence of LGE); (2) subendocardial LGE (diffuse subendocardial LGE with <50% transmurality in any short-axis images); (3) transmural LGE in more than half of the short-axis images.

### 2.4 Clinical follow-up

The primary endpoint was all-cause mortality. The patients were followed up in our center every 3–6 months by one of the investigators. The follow-up period was calculated as the time from the date of baseline CMR imaging to the date of the last available follow-up (March 2024) or death.

### 2.5 Statistical analysis

The data were summarized and analyzed using SPSS Statistics (version 26.0 International Business Machines, Inc., Armonk, New York,), GraphPad Prism 8.0 (GraphPad Software Inc., San Diego, California), and R (version 4.3.3, The R Project for Statistical Computing, Vienna, Austria). Data normality was assessed using the Kolmogorov-Smirnov test. Nominal variables were reported as absolute and frequency, and continuous variables were expressed as mean ± standard deviation or median (25% and 75% quartiles). In comparisons between survivors and non-survivors, the χ^2^ test was used for nominal variables, and for continuous variables, the independent samples t-test or the Mann-Whitney U test was used as appropriate. One-way analysis of variance or Kruskal-Wallis test with Bonferroni correction was used for comparison between the groups. Troponin T, NT-proBNP, and dFLC were log-transformed because of their skewed distributions. Survival was evaluated with Cox proportional hazards regression analysis, providing estimated hazard ratios (HR) with 95% confidence intervals (CI) and Kaplan-Meier curves. Median follow-up was calculated using the reverse Kaplan-Meier method. Univariate and multivariate Cox regression analysis was used to evaluate the independent prognostic factors among cohort variables and analyze their impact on overall survival. Multivariate Cox regression analysis (forward stepwise) using significant parameters of univariate analysis to identify independent predictors. Optimal cut-off value for risk stratification was calculated by optimizing the maximally selected log-rank test statistic. Survival analysis was performed using the Kaplan-Meier method and log-rank test. Statistical significance was defined as P < 0.05.

## 3 Results

### 3.1 Study population and clinical characteristics

A total of 126 patients with AL-CA amyloidosis were included. Table 1 summarizes the clinical, laboratory, and magnetic resonance parameters of the entire study cohort, survivors and non-survivors. Male patients were predominant (64%). The number of patients with Mayo stages I, II, III and IV were 16 (12.7%), 17 (13.5%), 32 (25.4%), and 61 (48.4%), respectively. Survivors had lower troponin T, NT-proBNP, dFLC, creatinine, and higher total cholesterol than those of the non-survivors. In addition, the LV mass and ECV of survivors were significantly reduced, but the stress MBF and MPR were higher. Compared with the survivors, the proportions of patients in the transmural LGE were higher in the non-survivors.

**Table 1:** Characteristics of the study population.

Characteristic	All patients (n = 126)	Survivors (n = 64)	Non-survivors (n = 62)	P value
*Basic and clinical data*				
Male sex, n (%)	81 (64)	40 (63)	41 (66)	0.671
Age, years	61.13±8.46	59.08±7.80	63.24±8.65	0.005
Height, cm	161.30±7.36	160.89±7.86	161.73±6.84	0.526
Weight, kg	58.38±10.11	58.01±9.29	58.76±10.96	0.680
BMI, kg/m^2^	22.35±3.06	22.32±2.62	22.37±3.47	0.924
Systolic blood pressure, mm Hg	103.00 (94.00; 115.75)	104.00 (96.00; 112.75)	102.00 (93.00; 119.25)	0.691
Diastolic blood pressure, mm Hg	69.00 (61.00; 79.00)	69.00 (62.00; 76.00)	69.00 (60.75; 81.25)	0.257
Smoking, n (%)	49 (38.9)	26 (40.6)	23 (37.1)	0.685
*Clinical chemistry*				
Troponin T, pg/mL	57.60 (31.53; 116.88)	36.00 (21.38; 64.85)	77.05 (53.75; 137.25)	<0.001
NT-proBNP, ng/L	4340.00 (1494.50; 9692.50)	1754.50 (688.75; 4053.75)	7666.00 (4620.50; 13,004.75)	<0.001
dFLC, mg/L	225.60 (63.78; 464.30)	82.95 (37.63; 257.45)	371.65 (192.10; 632.53)	<0.001
FBG, mmol/L	4.75 (4.20; 5.31)	4.80 (4.00; 5.37)	4.63 (4.20; 5.21)	0.321
Triglyceride, mmol/L	1.02 (0.81; 1.59)	1.09 (0.91; 1.64)	0.97 (0.74; 1.40)	0.141
Total cholesterol, mmol/L	3.82 (3.04; 4.51)	3.90 (3.24; 4.88)	3.65 (2.87; 4.15)	0.016
Creatinine, μmoI/L	84.40 (68.43; 110.03)	80.15 (64.23; 94.38)	94.70 (72.58; 140.25)	0.005
*CMR imaging parameters*				
LVEF, %	42.00 (31.50; 55.00)	45.00 (29.67; 60.75)	40.98 (33.50; 48.39)	0.077
LVESV, mL	58.70 (37.00; 81.23)	49.15 (34.09; 79.59)	67.87 (43.84; 82.74)	0.054
LVEDV, mL	104.00 (78.72; 124.52)	98.00 (76.75; 119.68)	111.86 (78.72; 133.52)	0.187
LVSV, mL	42.50 (28.96; 53.16)	43.00 (29.49; 55.89)	41.00 (28.67; 52.52)	0.648
Cardiac output, L/min	3.38 (2.54; 4.60)	3.50 (2.59; 4.28)	3.22 (2.37; 4.68)	0.899
LV mass, g	152.68 (120.08; 196.72)	137.53 (110.23; 187.73)	160.96 (128.92; 208.70)	0.029
LV wall thickness, mm	16.40 (14.08; 19.33)	15.45 (13.70; 18.73)	17.50 (14.83; 19.53)	0.076
Pre-T1, ms	1460.50 (1416.75; 1511.00)	1455.00 (1397.50; 1512.50)	1466.00 (1430.25; 1508.00)	0.253
ECV, %	55.40 (48.3; 60.23)	50.85 (43.98; 53.50)	60.00 (56.95; 64.23)	<0.001
Rest MBF, mL/min/g	0.70 (0.60; 0.83)	0.80 (0.63; 0.90)	0.70 (0.58; 0.80)	0.053
Stress MBF, mL/min/g	1.20 (0.88; 1.40)	1.30 (1.10; 1.50)	1.00 (0.80; 1.20)	<0.001
MPR	1.60 (1.50; 1.80)	1.70 (1.60; 1.80)	1.50 (1.40; 1.60)	<0.001
*LGE pattern*				< 0.001
No LGE, n (%)	11 (8.7)	9 (14.1)	2 (3.2)	
Subendocardial LGE, n (%)	56 (44.5)	39 (60.9)	17 (27.4)	
Transmural LGE, n (%)	59 (46.8)	16 (25.0)	43 (69.4)	
*Mayo stage 2012*				<0.001
Ⅰ, n (%)	16 (12.7)	16 (25.0)	0	
Ⅱ, n (%)	17 (13.5)	14 (21.9)	3 (4.8)	
Ⅲ, n (%)	32 (25.4)	18 (28.1)	14 (22.6)	
Ⅳ, n (%)	61 (48.4)	16 (25.0)	45 (72.6)	

Results are given as number (percentage) or mean ± standard deviation or median (25th; 75th percentile)

*BMI* body mass index, *dFLC* serum immunoglobulin free light chain difference, *ECV* extracellular volume fraction, *FBG* fasting blood glucose, *LVEDV* left ventricular end-diastolic volume, *LVESV* left ventricular end-systolic volume, *LVEF* left ventricular ejection fraction, *LVSV* left ventricular stroke volume, *LGE* late gadolinium enhancement, *MBF* myocardial blood flow, *MPR* myocardial perfusion reserve, *NT­-proBNP* N­-terminal pro-B-type natriuretic peptide, *LV* left ventricular

### 3.2 ECV, MPR, and cardiac involvement relationship

**Fig. 4:**
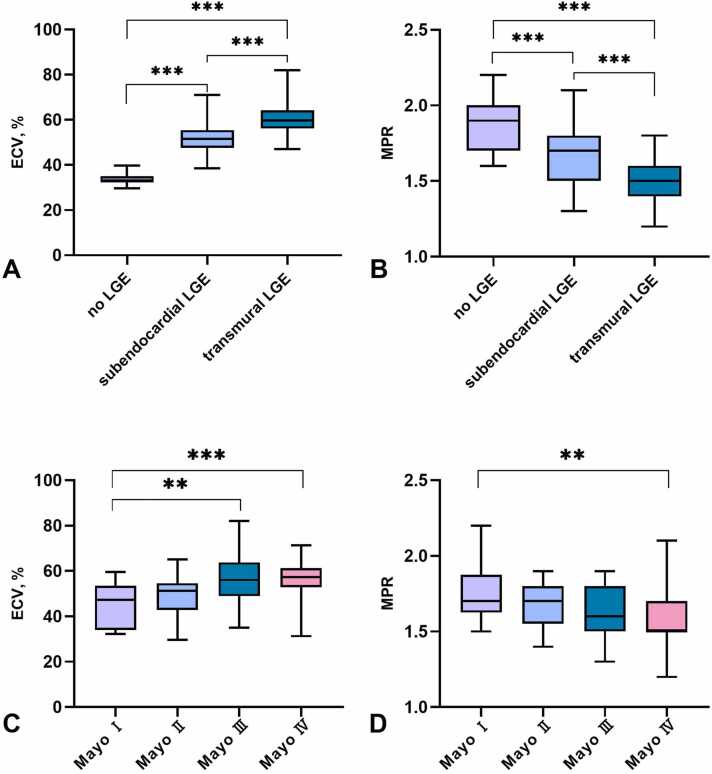
Relationship between ECV, MPR, and cardiac involvement. (A) Relationship between LGE pattern and ECV. (B) Relationship between LGE pattern and MPR. Both ECV and MPR were significantly decreased between no LGE and subendocardial LGE groups and between subendocardial LGE and transmural LGE groups and between no LGE and transmural LGE groups. (C) Relationship between the Mayo stages and ECV. (D) Relationship between the Mayo stages and MPR. The ECV progressively increases while the MPR gradually decreases as amyloidosis advances toward cardiac staging, as indexed by the Mayo 2012 staging system. *ECV* extracellular volume fraction, *LGE* late gadolinium enhancement, *MPR* myocardial perfusion reserve. **P < 0.01; ***P < 0.001

The ECV and MPR values in patients with different LGE patterns are shown in Fig. 4A and B. ECV of the transmural LGE group [59.80 (56.20–64.20)] was significantly higher than the no LGE [33.10 (32.30–35.00)] and subendocardial LGE groups [51.50 (47.50–55.45)] (P < 0.001). The MPR results for patients with no LGE, subendocardial LGE, and transmural LGE were 1.90 (1.70–2.00), 1.70 (1.50–1.80), and 1.50 (1.40–1.60) (P < 0.001), respectively. The MPR was significantly decreased between no LGE and subendocardial LGE groups, between subendocardial LGE and transmural LGE groups, and between no LGE and transmural LGE groups. In addition, the relationship between ECV, MPR, and cardiac involvement stages is presented in Fig. 4C and D. ECV of patients with Mayo stage Ⅳ was the highest, followed by patients with Mayo stage Ⅲ. MPR value was higher in patients with Mayo stage Ⅰ than in those with Mayo stage Ⅳ (P < 0.01).

### 3.3 Survival analysis

The median follow-up time was 37 months (95% CI: 33.6–40.4 months). A total of 62 (49%) patients died by the cut-off date. The results of univariate Cox regression analysis and the final multivariable Cox regression model resulting from variable selection are presented in Table 2. According to univariate Cox regression results, age (HR: 1.036, 95%CI: 1.005–1.068, P = 0.022), log(troponin T) (HR: 6.643, 95%CI: 3.147–14.020, P < 0.001), log(NT-proBNP) (HR: 16.991, 95%CI: 7.755–37.227, P < 0.001), log(dFLC) (HR: 2.594, 95%CI: 1.732–3.887, P < 0.001), ECV (HR: 1.100, 95%CI: 1.072–1.129, P < 0.001), stress MBF (HR: 0.277, 95%CI: 0.136–0.562, P < 0.001), MPR (HR: 0.001, 95%CI: 0.000–0.005, P < 0.001), and transmural LGE were associated with poor outcomes. In the final multivariate Cox regression model, only log(NT-proBNP) (HR: 4.037, 95%CI: 1.689–9.648, P = 0.002), log(dFLC) (HR: 1.988, 95%CI: 1.282–3.082, P = 0.002), ECV (HR: 1.081, 95%CI: 1.042–1.121, P < 0.001), MPR (HR: 0.002, 95%CI: 0.000–0.018, P < 0.001), and LGE were independent predictors.

**Table 2:** Univariable and Multivariable Cox regression analysis.

Parameter	Univariate Cox regression			Multivariate Cox regression		
	HR	95% CI	P value	HR	95% CI	P value
Male sex, n (%)	1.023	0.604–1.731	0.933			
Age, years	1.036	1.005–1.068	0.022			
Height, cm	1.007	0.973–1.041	0.702			
Weight, kg	1.001	0.975–1.027	0.962			
BMI	0.988	0.905–1.078	0.785			
Systolic blood pressure, mm Hg	1.002	0.987–1.016	0.813			
Diastolic blood pressure, mm Hg	1.013	0.992–1.034	0.242			
Smoking	0.859	0.513–1.439	0.564			
log(troponin T), pg/mL	6.643	3.147–14.020	<0.001			
log(NT-proBNP), ng/L	16.991	7.755–37.227	<0.001	4.037	1.689–9.648	0.002
log(dFLC), mg/L	2.594	1.732–3.887	<0.001	1.988	1.282–3.082	0.002
LVEF, %	0.986	0.971–1.001	0.071			
LVESV, mL	1.006	0.999–1.014	0.082			
LVEDV, mL	1.004	0.997–1.011	0.288			
LVSV, mL	0.995	0.982–1.009	0.482			
Cardiac output, L/min	0.965	0.817–1.140	0.672			
LV mass, g	1.002	1.000–1.005	0.098			
LV wall thickness, mm	1.039	0.971–1.111	0.273			
Pre-T1, ms	1.002	0.999–1.005	0.209			
ECV, %	1.100	1.072–1.129	<0.001	1.081	1.042–1.121	<0.001
Rest MBF, mL/min/g	0.376	0.093–1.520	0.170			
Stress MBF, mL/min/g	0.277	0.136–0.562	<0.001			
MPR	0.001	0.000–0.005	<0.001	0.002	0.000–0.018	<0.001
*LGE pattern*						
No LGE	\	\	\			
Subendocardial LGE	1.931	0.446–8.360	0.379	0.086	0.015–0.514	0.007
Transmural LGE	6.262	1.513–25.91	0.011	0.095	0.015–0.597	0.012

*BMI* body mass index, *CI* confidence interval, *dFLC* serum immunoglobulin free light chain difference, *ECV* extracellular volume fraction, *HR* hazard ratio, *LV* left ventricular, *LVEDV* left ventricular end-diastolic volume, *LVESV* left ventricular end-systolic volume, *LVEF* left ventricular ejection fraction, *LGE* late gadolinium enhancement, *LVSV* left ventricular stroke volume, *MBF* myocardial blood flow, *MPR* myocardial perfusion reserve, *NT­-proBNP* N­-terminal pro-B-type natriuretic peptide

### 3.4 Cut-off calculation and risk stratification

**Fig. 5:**
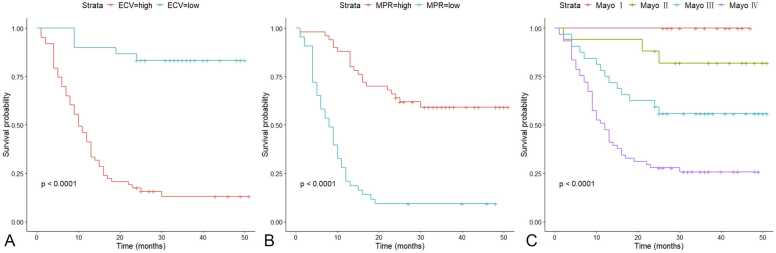
Kaplan-Meier plots of time to all-cause mortality. (A) Kaplan-Meier curves for all-cause mortality according to the cut-off value of ECV (53.6%). (B) Kaplan-Meier curves for all-cause mortality according to the cut-off value of MPR (1.5). Lower ECV and higher MPR were associated with better survival outcomes (all P < 0.001). (C) Kaplan-Meier curves for patients with diverse Mayo 2012 stages. The outcome of AL amyloidosis is strongly associated with the severity of cardiac involvement, patients with Mayo Ⅳ were significantly worse than patients with other Mayo stages. *ECV* extracellular volume fraction, *MPR* myocardial perfusion reserve

To investigate the clinical consequence of MPR, we divided patients into two groups relative to the MPR value, according to the cut-off determined by maximally selected log-rank statistics. The same method was used to obtain the optimal cut-off value for ECV. Corresponding Kaplan-Meier curves using the identified thresholds of MPR (1.5), ECV (53.6%), and Mayo stages for stratification are presented in Fig. 5. The ECV >53.6% and MPR ≤1.5 provided powerful discriminatory segregation curves for estimating survival in the entire cohort. Lower ECV and higher MPR were associated with better survival outcomes (all P < 0.001).

Based on the optimal threshold grouping, we analyzed the survival rates of different groups. In this study, the 1-, 2-, and 3-year survival rates in the MPR >1.5 group were 92.2%, 75.3%, and 70.9%, respectively. The 1-, 2-, and 3-year survival rates of MPR ≤1.5 were 28.6%, 18.4%, and 18.4%, respectively. Furthermore, the survival rates of ECV >53.6% were progressively decreased, with 45.7% for 1 year, 24.3% for 2 years, and 18.9% for 3 years. And the patients with ECV ≤53.6% had 1-, 2-, and 3-year survival rates of 94.6%, 89.3%, and 89.3%, respectively.

**Fig. 6:**
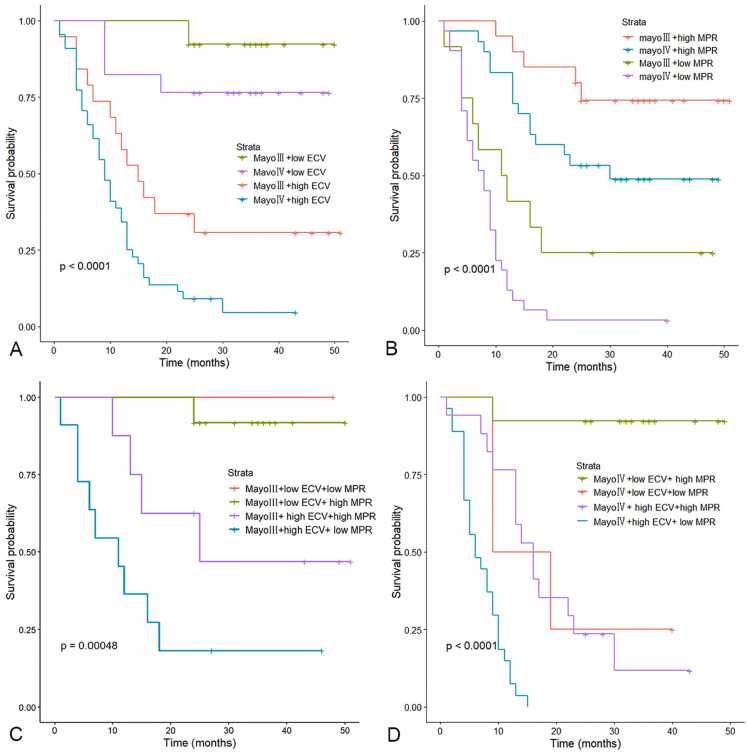
Risk stratification based on the new scoring system incorporating ECV, MPR, and Mayo 2012 stages. The thresholds were 53.6% for ECV and 1.5 for MPR. (A) Kaplan-Meier survival curve for ECV based on Mayo stages (Ⅲ or Ⅳ). (B) Kaplan-Meier survival curve for MPR based on Mayo stages (Ⅲ or Ⅳ). Survival was significantly worse in the presence of ECV >53.6%, and in the presence of MPR ≤1.5. In addition, both ECV and MPR further differentiated patient survival in the presence of the same Mayo staging. (C and D) A new risk stratification system combined ECV and MPR with Mayo stage Ⅲ or Ⅳ. In patients with higher ECV of Mayo stages (Ⅲ or Ⅳ), the patients with higher MPR had higher survival. Survival curves were compared using log-rank test. *ECV* extracellular volume fraction, *MPR* myocardial perfusion reserve

We then built new assessment models that incorporated ECV and MPR into the Mayo staging system to determine the predictive incremental value of the imaging parameters. After adding ECV and MPR, we found that the differences in survival probability were significant among patients at Mayo stages Ⅲ and Ⅳ (Fig. 6). Kaplan-Meier survival analysis showed significantly increased mortality in the presence of ECV >53.6% (P < 0.001), and in the presence of MPR ≤1.5, which was irrespective of Mayo stages (Fig. 6A and B). In patients with higher ECV of Mayo stage Ⅲ, the patients with higher MPR had higher survival compared with patients with lower MPR (Fig. 6C). This trend remained to be significant in subgroups of patients with advanced Mayo stage Ⅳ (Fig. 6D).

## 4 Discussion

This study sought to investigate the extent of microvascular dysfunction and its incremental prognostic value in AL-CA by quantitative stress perfusion. (1) With the progression of Mayo staging and increased cardiac involvement, MPR gradually decreases while ECV increases. (2) ECV, MPR, and LGE were independently associated with survival. Moreover, the integration of ECV and MPR allowed for a more nuanced stratification of survival outcomes in patients with Mayo Ⅲ or Ⅳ. (3) MPR, a quantitative stress perfusion CMR parameter, was an independent prognostic predictor of mortality in AL-CA patients and provided incremental prognostic value, which can further differentiate patient survival even when Mayo staging and ECV were identical.

Increased ventricular mass from cardiac amyloid deposition may impede myocardial perfusion due to vascular rarefaction and compression, resulting in myocardial microvascular dysfunction. An elevation in left ventricular myocardial mass is frequently correlated with a diminished MPR. Consistent with previous studies, our pathologic finding indicated that amyloid deposits in the myocardial interstitium and perivascular regions enlarged the extracellular space of the myocardium, leading to capillary rarefaction and extravascular compression, which may explain an increase in ECV [16].

Our study revealed that stress MBF and MPR were lower in deceased patients compared with survivors, suggesting that myocardial microvascular dysfunction was associated with the risk of mortality in AL-CA. This finding ties well with the previous observation by Dorbala et al., who found a significant decrease in MBF and coronary flow reserve in CA patients who underwent evaluation of coronary microvascular function with positron emission tomography/computed tomography [17]. Microvascular ischemia resulting from intramyocardial coronary artery obstruction by amyloid deposition can be a probable cause of abnormal blood flow reserve [18]. In addition, myocardial amyloid deposition leads to cardiomyocyte necrosis and interstitial fibrosis [19], and fibrotic tissue remodeling after injury may lead to further functional impairment and poor outcomes. Myocardial fibrosis can trigger extracellular matrix remodeling, leading to fibrotic scar formation that increases myocardial mass, which contributes to intramyocardial microvascular rarefaction and reduced capillary density. Higher LV mass was related to microvascular dysfunction, as well as to reduced stress MBF and reduced MPR. These results further support the conclusion that coronary microvascular dysfunction is a major cause of myocardial ischemic injury in patients with amyloidosis.

This study compared ECV between subgroups of different LGE patterns, suggesting that ECV correlates with the degree of cardiac involvement from amyloid deposition. Transmural LGE appears to be the pattern that carries the most adverse prognosis, which is consistent with the findings of Fontana et al. [20]. In addition, our study indicated significant differences in MPR between different LGE patterns. The MPR in the transmural LGE pattern was significantly lower than those in both the subendocardial LGE and no LGE patterns, providing a basis for using quantitative magnetic resonance blood flow parameters to assess the extent of cardiac involvement. The more extensive the infiltration of myocardial amyloid and the more severe the cardiac involvement, the lower the myocardial flow reserve of the patients. This reaffirms the point that myocardial microcirculation may reflect amyloid infiltration and potentially affect prognosis.

The recently published ASNC/AHA/ASE/EANM/HFSA/ISA/SCMR/SNMMI Expert Consensus Recommendations emphasized the diagnostic and prognostic value of CMR in myocardial amyloidosis [21]. CMR can be invaluable for quantifying cardiac amyloid burden and making prognostic prediction, with good reproducibility for tracking changes over time or with treatment. So far, the prognostic value of myocardial perfusion parameters obtained by quantitative perfusion CMR in AL patients with biopsy-confirmed cardiac involvement has not been studied. We found that the stress MBF and MPR were decreased significantly in the non-survivors compared with the survivors. The findings of our study indicated that MPR significantly contributes to risk stratification and prediction of outcomes among patients with AL cardiac amyloidosis. Our findings validated that MPR is an independent predictor of survival, offering additional prognostic value. Multiple studies have shown that reduced MPR was associated with an increased risk of cardiovascular events [22]. It has been suggested that there are biases and systematic errors in the stress and rest MBF, which are eliminated when measuring MPR [23]. Thus, MPR may be superior to stress MBF in predicting death. CMR can be used as a useful non-invasive tool for monitoring disease progression and predicting prognosis in patients with cardiac amyloidosis by assessing myocardial blood flow. Clearance of amyloid deposits will also improve myocardial perfusion, making myocardial perfusion a significant marker of treatment response [12].

## 5 Limitations

Our study has several limitations. First, because this study was conducted at a single center, additional multicenter studies are warranted to confirm the findings. Second, the diagnosis of AL amyloidosis is often delayed due to the heterogeneity of symptoms at presentation. The onset and true duration of cardiac involvement in AL amyloidosis are challenging to assess because patients often exhibit no clinical symptoms even in the beginning of cardiac involvement. Most patients are asymptomatic until a late stage of disease. Patients in our cohort may have had varying disease severity. In addition, the response to different therapeutic strategies may vary among patients with different CMR and clinical variables, which was not investigated in the study. Finally, the cut-off value identified in the present study may not be applicable when using a different system and must be considered in the context of the study population.

## 6 Conclusions

The progression of Mayo stage correlates with a decrease in MPR and an elevation in ECV. ECV and MPR are independently associated with all-cause mortality in the AL-CA patients. MPR may serve as a new imaging predictor of cardiac involvement and provide an incremental prognostic value in AL amyloidosis.

## Funding

This study was supported by the 10.13039/501100001809National Natural Science Foundation of China (No. 81701660) and 10.13039/501100004735Natural Science Foundation of Hunan Province (No. 2020JJ5842).

## Author contributions

Lin Tian: Software, Resources, Methodology. Kang Li: Methodology, Investigation, Data curation. Hu Guo: Software, Resources. Xiaoyue Zhou: Software, Resources. Mu Zeng: Writing—review and editing, Validation, Supervision, Methodology, Funding acquisition, Formal analysis, Data curation, Conceptualization. Leting Tang: Writing—review and editing, Writing—original draft, Visualization, Validation, Software, Resources, Methodology, Formal analysis, Data curation, Conceptualization. Wenjin Zhao: Methodology, Investigation, Formal analysis.

## Ethics approval and consent

This study was approved by the ethics committee of the Second Xiangya Hospital of Central South University, and informed consent was provided by all participants (LYF2023027). Finally, all the procedures were conducted in accordance with relevant guidelines and regulations.

## Consent for publication

All patients provided written informed consent.

## Declaration of competing interests

The authors declare that they have no known competing financial interests or personal relationships that could have appeared to influence the work reported in this paper.

## Data Availability

All data are available from the corresponding author on request.
